# Meningitis Caused by *Streptococcus suis* Serotype 14, North America

**DOI:** 10.3201/eid1502.080842

**Published:** 2009-02

**Authors:** Ahmed Haleis, Michelle Alfa, Marcelo Gottschalk, Kathryn Bernard, Allan Ronald, Kanchana Manickam

**Affiliations:** University of Manitoba, Winnipeg, Manitoba, Canada (A. Haleis, A. Ronald); St. Boniface General Hospital, Winnipeg (M. Alfa, K. Manickam); University of Montreal, St.-Hyacinthe, Quebec, Canada (M. Gottschalk); National Microbiology Laboratory, Winnipeg (K. Bernard)

**Keywords:** Streptococcus suis serotype 14, meningitis, occupational zoonosis, human infections, letter

**To the Editor:** *Streptococcus suis* is an opportunistic pathogen that can cause serious systemic infections in pigs and occupation-related infections in humans who work in close contact with pigs or pork by-products. Most *S. suis* organisms isolated from diseased pigs belong to serotypes 1–8 (*1*). The most prevalent strain worldwide is serotype 2, which causes invasive infections in pigs and humans (*2*). We report a case of human meningitis caused by *S. suis* serotype 14.

The patient was a 59-year-old woman from rural Manitoba, Canada; she worked at a hog plant and handled 300–400 piglets/day. In October 2007, when she sought care, she had a 2-day history of fever, vomiting, headache, neck pain, and reduced consciousness. She was febrile and confused and had meningeal signs. Leukocyte count was 19,900/mm^3^. Cerebrospinal fluid (CSF) had 284 × 10^6^/L leukocytes (59% lymphocytes, 41% polymorphonuclear cells), 2.3 mmol/L glucose, and 1.85 g/L total protein. Gram stain of CSF showed gram-positive cocci in pairs; cefotaxime and vancomycin were prescribed empirically. Results of computed tomography of the head, chest radiograph, and transesophageal echocardiogram were within normal limits. Blood culture was negative after 5 days of incubation. The CSF culture grew small α-hemolytic colonies on blood agar and chocolate agar. The organisms were gram-positive cocci in chains, were catalase negative, and were identified as *S. suis* by Vitek II and API 20 Strep System (both from bioMérieux, St.-Laurent, Quebec City, Canada).

Identification of the organism as *S. suis* was confirmed at the National Microbiology Laboratory, Winnipeg, Manitoba, Canada, by conventional biochemical tests (*3*), the results of which were consistent with that of the type strain (Table) and were also confirmed by 16S rRNA gene sequencing, which showed 100% homology with the *S. suis* type strain ATCC 43765, GenBank accession no. EU 477176.

**Table:** Identification of organism isolated from cerebrospinal fluid of 59-year-old woman with meningitis, Manitoba, Canada*

Test	*Streptococcus suis* (*3*)	Patient isolate
α-hemolysis on sheep blood agar	+	+
Motility	–	–
Catalase	–	–
Oxidase	ND	–
Fermented		
l-arabinose	–	–
d-glucose	+	+
Glycerol	–	–
Inulin	+	+
Lactose	+	+
Maltose	+	+
Mannitol	–	–
Melezitose	–	–
Melibiose	Variable	+
Raffinose	Variable	+
Ribose	–	–
Salicin	+	+
Sorbitol	–	–
Sucrose	+	+
Trehalose	+	+
Hydrolyzed		
l-arginine	+	–
Esculin/bile esculin	+/ND	±
Starch	+	+
Glycogen	+	+
Hippurate	–	–
Acetoin	–	–
Optochin disk	Resistant	ND
Enzymes		
α-galactosidase	+	+
β-galactosidase	Variable	+
β-glucuronidase	+	+
Leucine arylamidase	+	+
N-acetylglucosaminidase	+	+
Acid phosphatase	–	–
Alkaline phosphatase	–	–
Pyrrolidonylarylamidase	–	–
API Strep code		4640473 high degree (97%) confidence *S. suis*

*+, positive; –, negative; ND, not done; API Strep code, API 20 Strep, API System (bioMérieux, St.-Laurent, Quebec City, Canada).

Antimicrobial-drug susceptibilities were determined by microbroth dilution by using Sensititre STP3F panels (Nova Century Scientific Inc., Burlington, Ontario, Canada) and cation-adjusted Mueller Hinton broth with lysed horse blood (2%–5% vol/vol) by TREK Diagnostic Systems, Inc. (Nova Century Scientific Inc.) using manufacturer's instructions and following Clinical and Laboratory Standards Institute guidelines for *Streptococcus* spp. other than *S. pneumoniae* (*4*). This isolate was sensitive to penicillin, cefepime, cefotaxime, ceftriaxone, linezolid, trimethoprim and sulfamethoxazole, vancomycin, meropenem, and levofloxacin; it was resistant to azithromycin, erythromycin, and tetracycline. The isolate was sent to the International Reference Laboratory at the Université de Montréal, Montréal, Québec, Canada, for *S. suis* serotyping, where it was identified by the coagglutination test as serotype 14 (*5*).

The patient recovered quickly, and her therapy was changed to penicillin G. She was transferred to her local hospital to complete her medication. Within a week of her initial visit, bilateral deafness and loss of balance developed and progressed over the next month and had not ameliorated after 1 year.

Human *S. suis* infections result primarily from direct contact (with wounds on skin or mucosa of the mouth and nasal cavity) with carrier pigs, sick pigs, or raw pork contaminated with *S. suis* (*2*). The infection rate among abattoir workers, pig breeders, meat processing workers, and veterinarians is ≈3 cases/100,000 (1,500× higher than the rate for the general population) (*2*). A striking sequela to *S. suis* meningitis is deafness or vestibular dysfunction (*2*,*6*). A consistently higher percentage of persons experiences deafness after *S. suis* infection than after infection with other meningitis-causing bacteria, 50% and 65% in Europe and Asia, respectively (*2*).

Most cases of *S. suis* infection in humans have been attributed to serotype 2 strains. Only 4 human cases have been reported in North America: 2 in Canada (1 endocarditis, 1 meningitis) and 2 cases of meningitis in the United States (*6*–*9*). All 4 cases were attributed to *S. suis* serotype 2. Serotype 14 has been reported as a human pathogen in the Netherlands, Thailand, the United Kingdom, and Denmark and has been routinely isolated from diseased pigs in Canada (*10*).

Although in pigs the organism is present in the upper respiratory tract, particularly the tonsils, nasal cavities, genital tract, and alimentary tract, the mode of transmission to humans reported so far had been through cuts in the hands. Our patient handled hundreds of piglets every day and most likely acquired the infection through her hands. Her meningitis was complicated by bilateral hearing loss with vestibular dysfunction. Preexisting medical conditions, such as alcoholism, liver cirrhosis, or splenectomy, have been described to predispose patients to severe infection and hearing loss (*2*). Our patient, however, did not have any predisposing conditions.

Meningitis in humans caused by *S. suis* serotype 14 is less common than that caused by serotype 2, but the consequences are similar and can be reduced by early treatment with antimicrobial drugs. Identifying this case of meningitis caused by *S. suis* serotype 14 in Canada raises concerns about the public health aspect of this infection. Guidelines may be required to ensure that staff working in hog plants are aware of the risk for this infection and that they use adequate personal protective equipment.
